# Assessing spatiotemporal trends of total and extreme precipitation in a subtropical highland region: A climate perspective

**DOI:** 10.1371/journal.pone.0289570

**Published:** 2023-08-04

**Authors:** Abdulnoor Ali Jazem Ghanim, Muhammad Naveed Anjum, Ghulam Rasool, Saifullah, Muhammad Irfan, Saifur Rahman, Salim Nasar Faraj Mursal, Usama Muhammad Niazi

**Affiliations:** 1 Civil Engineering Department, College of Engineering, Najran University, Najran, Saudi Arabia; 2 Department of Land and Water Conservation Engineering, Pir Mehr Ali Shah Arid Agriculture University Rawalpindi, Rawalpindi, Pakistan; 3 Electrical Engineering Department, College of Engineering, Najran University, Najran, Saudi Arabia; 4 Department of Mechanical Engineering Technology, National Skills University Islamabad, Islamabad, Pakistan; Arab Academy for Science Technology and Maritime Transport, EGYPT

## Abstract

This study used a dataset of 30 years (1990–2020) of daily observations from 24 meteorological stations in the northern highlands of Pakistan to assess trends in extreme precipitation indices. The RClimDex model was used to analyze the indices, and the Modified Mann-Kendal test and the Theil-Sen slope estimator were applied to determine trends and slopes, respectively. The results showed a significant decrease in total annual precipitation amount (PRCPTOT) with varying rates of negative trend from -4.44 mm/year to -19.63 mm/year. The total winter and monsoon precipitation amounts were also decreased during the past three decades. The intensity-based precipitation indices (RX1Day, RX5Day, R95p, R99p, and SDII) showed a significant decrease in extreme intensity events over time, while the count of consecutive dry days (CDD) and consecutive wet days (CWD) indicated a significant decrease in duration at multiple stations. The annual counts of days with precipitation more than or equal to 10 mm (R10), 20 mm (R20), and 25 mm (R25) exhibited a significant decrease in frequency of extreme precipitation events, with the decrease more pronounced in the northern parts of the study domain. The findings of this study indicate a significant decline in the intensity, frequency, and extent of precipitation extremes across the northern highlands of Pakistan over the past 30 years.

## 1. Introduction

Precipitation is one of the most important components of the hydrological cycle, which displays a wide range of spatio-temporal variability across the entire world. Changes in precipitation can significantly affect human life and the natural environment [1]. The intensity and frequency of precipitation extremes have been shifting, notably under anthropogenically driven climate warming, which has become increasingly obvious over the past few decades [2, 3]. Extreme precipitation events can significantly impact a region’s hydrological regime and socio-economic well-being [4, 5]. The occurrence of extreme precipitation events can have devastating impacts, including the potential for flooding and landslides, which can result in significant damage to infrastructure and transportation and communication networks, as well as loss of life [6].

Mountains are a key component of the Earth’s climate system and are particularly susceptible to the adverse impacts of climate change [7]. These regions act as water towers, delivering freshwater to communities downstream, and are frequently distinguished by complicated terrain that affects precipitation patterns [1]. Changes in precipitation patterns can have a significant impact on water supplies, ecosystems, and socioeconomic systems. Evaluating the effects of climate change, identifying potential dangers, and establishing appropriate adaptation measures all require an understanding of the historical trends and variability of total and extreme precipitation in mountainous regions. Research on historical trends in total and extreme precipitation occurrences in mountainous places, particularly in subtropical highland regions like the northern highlands of Pakistan, is, however, limited.

In Pakistan, the agricultural industry relies heavily on the dependable and plentiful water supply provided by precipitation [8]. Changes in precipitation would have serious consequences on the country’s agricultural output [9]. Severe extreme precipitation occurrences in Pakistan can have a significant effect on the region’s hydrological regimes and socioeconomic wellbeing [10]. These events are caused by the convergence of moist air from the Arabian Sea and the Bay of Bengal and the orographic lifting of the air as it passes over the Himalayas and the Hindu Kush mountain ranges [11]. The northern highlands of Pakistan (NHP) are particularly vulnerable to these extreme rainfall events due to the mountainous terrain and increasing human intervention in the region [7, 12]. Extreme precipitation events are predicted to become more intense and frequent as a result of climate change, which is thought to amplify these impacts in the future [13, 14].

Several investigations have been performed to evaluate the changes in extreme precipitation events across different regions globally [11, 15–18]. Despite a general trend of rising extreme precipitation events, there is a marked spatial and temporal variability in the frequency and intensity of such events across climatic regions [19]. In more recent studies, an increasing trend in extreme precipitation events has been described over the Tibetan Plateau [15, 20], South Asia [16], Central Asia [21, 22], and Europe [23]. Mainland China has also seen an increase in the frequency and intensity of extreme precipitation events over the past few decades [24]. Bangladesh has also recorded an upward trend in extreme precipitation events [25]. However, a decreasing trend in event-based extreme precipitation of various return periods has been noted in Pakistan [26, 27]. Based on the findings of preciously available literature, it appears that climate change has increased both the temporal and spatial variability of the occurrences of precipitation extremes across the world.

Extreme precipitation events in Pakistan can have serious consequences for the country’s economy and population, making it crucial to examine how these events have changed through time and space [28]. Flooding and other forms of extreme precipitation can cause infrastructure damage, disrupt transportation and communication networks, and ultimately result in human casualties and material losses. Additionally, extreme precipitation events can exacerbate water scarcity and lead to food insecurity, particularly in rural areas where agriculture is a main source of livelihood. To minimize the adverse effects of extreme precipitation events, it is critical to understand the frequency and intensity of these events, as well as their probable causes and implications. Although some previous studies have assessed the changes in the extreme precipitation events in Pakistan [26, 29–31], no particular study was conducted to investigate the changes in the precipitation extreme events in the NHP. Under this context, this study aims to investigate the spatiotemporal changes in the patterns of extreme precipitation events in the NHP over the recent past three decades. The study distinguishes itself through its utilization of an extended temporal coverage, and the incorporation of additional extreme indices. Findins of this assessment are anticipated to provide significant insights to the government and non-governmental organizations involved in enhancing disaster risk management and developing adaptation policies in the country.

## 2. Materials and methodology

### 2.1. Study region and datasets

**Fig 1:**
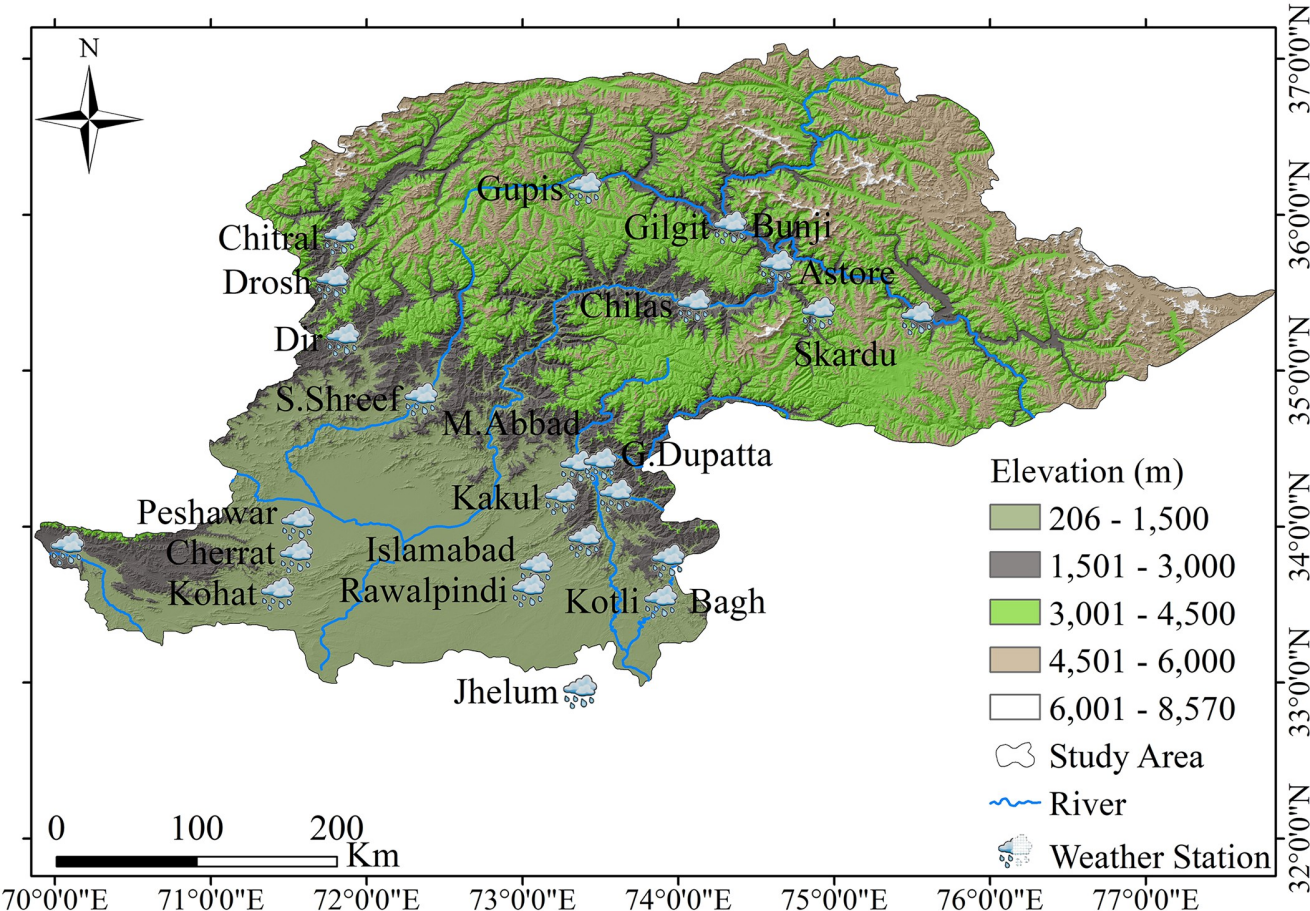
Location map of the study domain and considered stations.

Northern Pakistan encompasses a diverse range of landscapes, including high mountain ranges, plateaus, and valleys [26]. This region is characterized by its unique topography and varied climate patterns, which play an vital role in shaping the local environment and its natural resources. The northern highlands of Pakistan (covers spatial domain 33–37° N and 70–78° E, Fig 1) are one of the most notable features of this region and are the focus of this study. The northern highlands of Pakistan (NHP) encompass the Hindu-Kush, Karakoram, and Himalayas Mountain ranges. These mountain ranges are some of the highest in the world, with peaks reaching over 8,000 meters above sea level. The NHP are also home to several high-altitude glaciers and snow-covered peaks, which have a significant role in the hydrology and water supply of the region [31].

**Fig 2:**
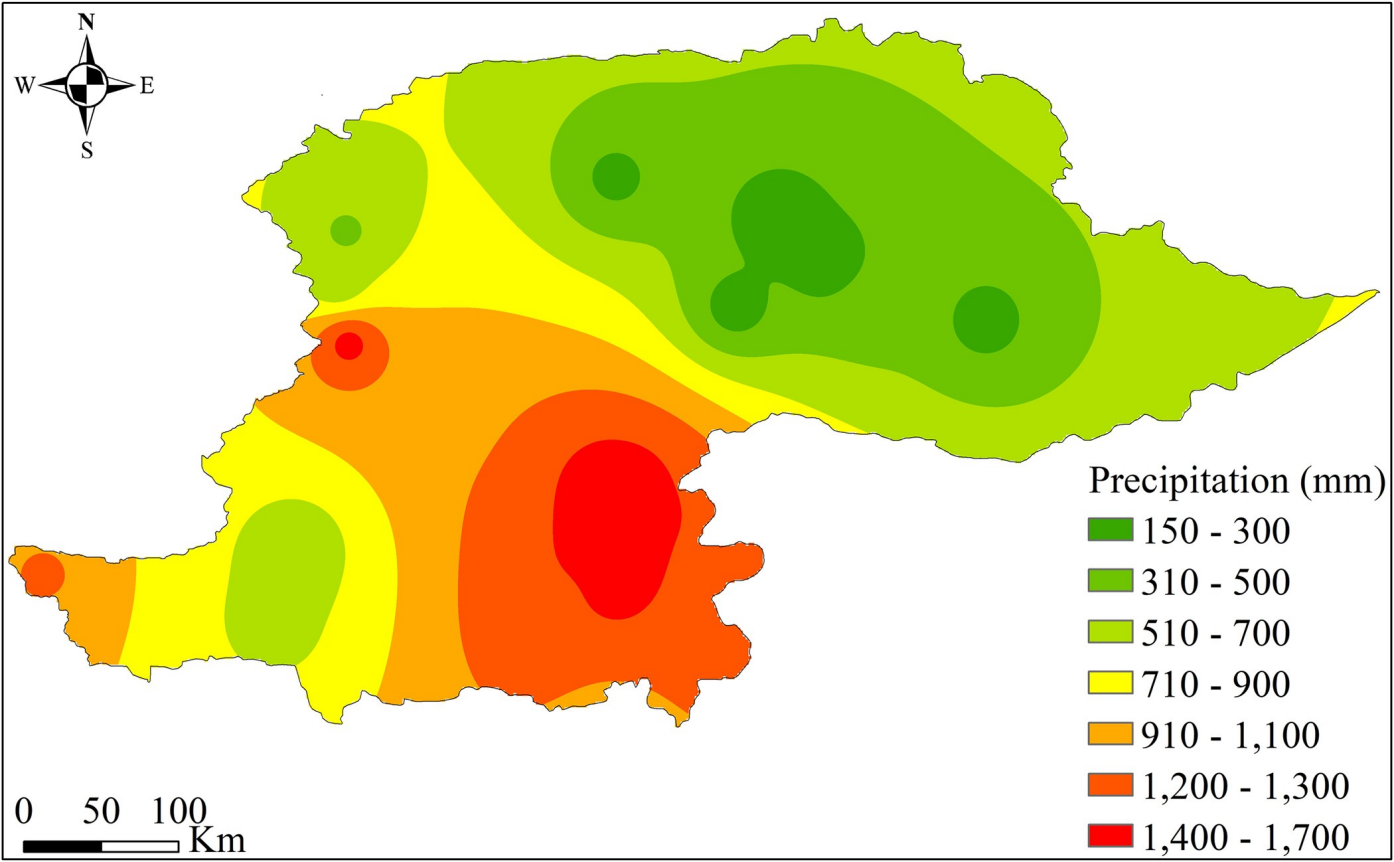
Spatial variability of average annual precipitation over the NHP.

The climate of the NHP is characterized by cold and dry winter and hot and humid summer months. The region experiences heavy snowfall and rainfall during the winter months, contributing to its snowmelt runoff and its water resources replenishing. The summer months are typically characterized by monsoon rains, which are important for the region’s agriculture and water resources. In the NHP, the precipitation is highly heterogeneous in its spatial and temporal distribution. The yearly average precipitation varies significantly, with values ranging from 145 mm to 1650 mm, following a southeast to northeast gradient (Fig 2).

This region plays an important role in shaping the local environment and its natural resources. It is a critical area of study for understanding the spatiotemporal trends of extreme rainfall events and their impacts on the region and its inhabitants. The purpose of this study is to investigate how extreme precipitation occurrences have changed over the course of the previous three decades (1990–2020) in terms of both their spatial and temporal patterns. For this purpose, daily precipitation records of 23 meteorological stations were obtained from the Pakistan Meteorological Department (PMD), whereas data of one station (Bagh) was obtained from the Water and Power Development Authority (WAPDA) of Pakistan. The selection of station data was done under relatively strict guidelines in order to assess extreme climate indices. This study adheres to the following standards: (i) a monthly data set is considered complete if it has no more than five missing days; (ii) a yearly data set is regarded complete if all of its months are complete as per the aforementioned criterion (i); and (iii) a station’s data is declared complete if it has no more than five missing years in its entire record [32, 33]. PMD has already assured the quality and homogeneity of the records. Table 1 shows the study area’s elevation and locations of the considered meteorological stations.

**Table 1:** Locations and elevations of meteorological stations considered in this study.

Sr. No.	Station	Lat (dd)	Long (dd)	Elevation (m)	Average Annual Precipitation (mm)
1	Astore	35.36	74.91	2168	460
2	Bagh	33.78	74.09	2200	1166
3	Balakot	34.38	73.35	981	1475
4	Bunji	35.67	74.63	1470	161
5	Cherrat	33.81	71.55	1372	663
6	Chilas	35.42	74.10	1251	197
7	Chitral	35.84	71.82	1500	466
8	Dir	35.21	71.86	1370	1365
9	Drosh	35.56	71.77	1465	549
10	Gari Dupatta	34.20	73.60	814	1339
11	Gilgit	35.92	74.33	1457	146
12	Gupis	36.17	73.40	2156	249
13	Islamabad	33.73	73.09	569	1263
14	Jhelum	32.94	73.37	287	862
15	Kakul	34.18	73.25	1309	1312
16	Kohat	33.57	71.43	513	616
17	Kotli	33.52	73.89	614	1184
*18*	Muzafarabad	*34*.*40*	*73*.*50*	*2303*	*1378*
19	Murree	33.91	73.40	2127	1695
20	Parachinar	33.86	70.08	1726	1115
21	Peshawar	34.02	71.56	360	530
22	Rawalpindi	33.59	73.04	540	1249
23	Saidu Shrif	34.82	72.35	970	1037
24	Skardu	35.34	75.54	2316	228

### 2.2. Precipitation indices

Each station’s extreme precipitation was analyzed using eleven indices developed by the “Expert Team on Climate Change Detection and Indices (ETCCDI)” project and endorsed by the “Commission for Climatology (CCl)” of the “World Meteorological Organization (WMO)” and the “Climate Variability and Predictability (CLIVAR)” program of the “World Climate Research Programme (WCRP)” [34]. RClimDex software developed by the ETCCDI was used to process the data. Bootstrapping was used to determine values for the time series of the very wet day (R95p) and extremely wet day (R99p) percentile-based indices, ensuring that there is no break in the data series of the indices at the start or end of the study duration [34].

The 11 ETCCDI-based extreme precipitation indices, considered for this study, are defined in Table 2. The PRCPTOT, R95p, R99p, maximum 1-day precipitation (RX1Day), maximum 5-day precipitation (RX5day), and simple daily intensity index (SDII) indices characterize the magnitude of intense rainfall events. The frequency of annual rainfall occurrences of varying intensities was evaluated using the R10, R20, and R25 indices. The lengths of the driest and wettest times of the year were assessed using the CDD and CWD indices (consecutive dry and wet days), respectively.

**Table 2:** The 11 ETCCDI-based extreme precipitation indices examined in this analysis and their respective definitions.

Sr. No.	Index	Definitions	Symbol
1	“Total annual precipitation”	"Total annual precipitation of rainy days (RR≥1 mm)"	PRCPTOT
2	“Total winter season precipitation”	"Total winter season precipitation of rainy days (RR≥1 mm)"	WPRCPTOT
3	“Total monsoon season precipitation”	"Total monsoon season precipitation of rainy days (RR≥1 mm)"	MPRCPTOT
4	“Consecutive dry days”	"Maximum number of consecutive days with RR<1 mm"	CDD
5	“Consecutive wet days”	"Maximum number of consecutive days with RR≥1 mm"	CWD
6	“Simple daily intensity index”	"Annual precipitation total divided by the number of annual rainy days (RR≥1 mm)"	SDII
7	“Number of heavy precipitation days”	"Annual counts of days when PRCP≥10 mm"	R10
8	“Number of heavy precipitation days”	"Annual counts of days when PRCP≥20 mm"	R20
9	“Number of heavy precipitation days”	"Annual counts of days when PRCP≥25 mm"	R25
10	“Very wet day precipitation”	"Annual precipitation total of RRN95th percentile"	R95p
11	“Extremely wet day precipitation”	"Annual precipitation total of RRN99th percentile"	R99p
12	“Maximum 1-day precipitation”	"Monthly maximum 1-day precipitation"	RX1day
13	“Maximum 5-day precipitation”	"Monthly maximum 5-day precipitation"	RX5day

### 2.3. Trends analysis

The linear trends in precipitation indexes were determined using a non-parametric Modified Mann-Kendall (MMK) Test. This approach is more resistant to the impact of outliers in a series and works better with data with non-normal distributions. The investigation included an examination of the trends in extreme precipitation indices on a daily basis. Total precipitation patterns were also analyzed at yearly and seasonal (winter and monsoon) timescales. Spatial variability of extreme precipitation events in the research area was assessed based on trend analysis results of each gauging station.

The MMK test is a modification of the Mann-Kendal test. The Mann-Kendall test was modified by Hamed and Rao in 1998 to account for the corrected variance of the Mann-Kendall statistics S [35]. Equations for calculating S, standardized test statistic Z, variance V(S), and corrected variance V*(S) are provided as below:

$$S=\sum_{j=1}^{n-1}\sum_{k=j+1}^{n}\mathrm{sgn}(x_{k}-x_{j})$$ (1)

$$sgn(x_{k}-x_{j})=\left\{\begin{array}{c} 1\;if\;x_{k}-x_{j}>0\\ 0\;if\;x_{k}-x_{j}=0\\ -1\;if\;x_{k}-x_{j}<0 \end{array}\right\}$$ (2)

$$V(S)=[n(n-1)(2n+5)-\sum_{t}t(t-1)(2t+5)/18]$$ (3)

V*(S)=V(S)Cor (4)

where,

$$Cor=1+\frac{2}{n(n-1)(n-2)}\sum_{i=1}^{n-1}(n-1)(n-i-1)(n-i-2)\rho_{s}(i)$$ (5)

$$Z=\left\{\begin{array}{c} \frac{S-1}{\sqrt{V}(S)}\;if\;S>0\\ 0\;if\;S=0\\ \frac{S+1}{\sqrt{V}(S)}\;if\;S<0 \end{array}\right\}$$ (6)

where *x_j_* and *x_k_* denote the values in the time-series, with k being greater than j. t is the span of time, n is the number of observations, and *ρ_s_*(*i*) is the significant autocorrelation function of the observations’ ranks. If Z is positive, it shows an increasing trend, whereas a negative value indicates a decreasing trend. The trend’s significance was determined at a 5% level of significance.

In order to get an accurate assessment of the slope of the trend, Theil-Sen (TS) approach was used. The slope, according to TS, was calculated as follows:

$$T_{i}=\frac{x_{j}-x_{k}}{j-k}$$ (7)

where *x_j_* and *x_k_* indicate the data values at times j and k, respectively.


$$Q_{i}=\left\{\begin{array}{l} T_{\frac{N+1}{2}}\;N\;is\;odd\\ \frac{1}{2}(T_{\frac{N}{2}}+T_{\frac{N+2}{2}})\;N\;is\;even \end{array}\right\}$$ (8)

## 3. Results

### 3.1. Analysis of annual and seasonal total precipitation trends

**Fig 3:**
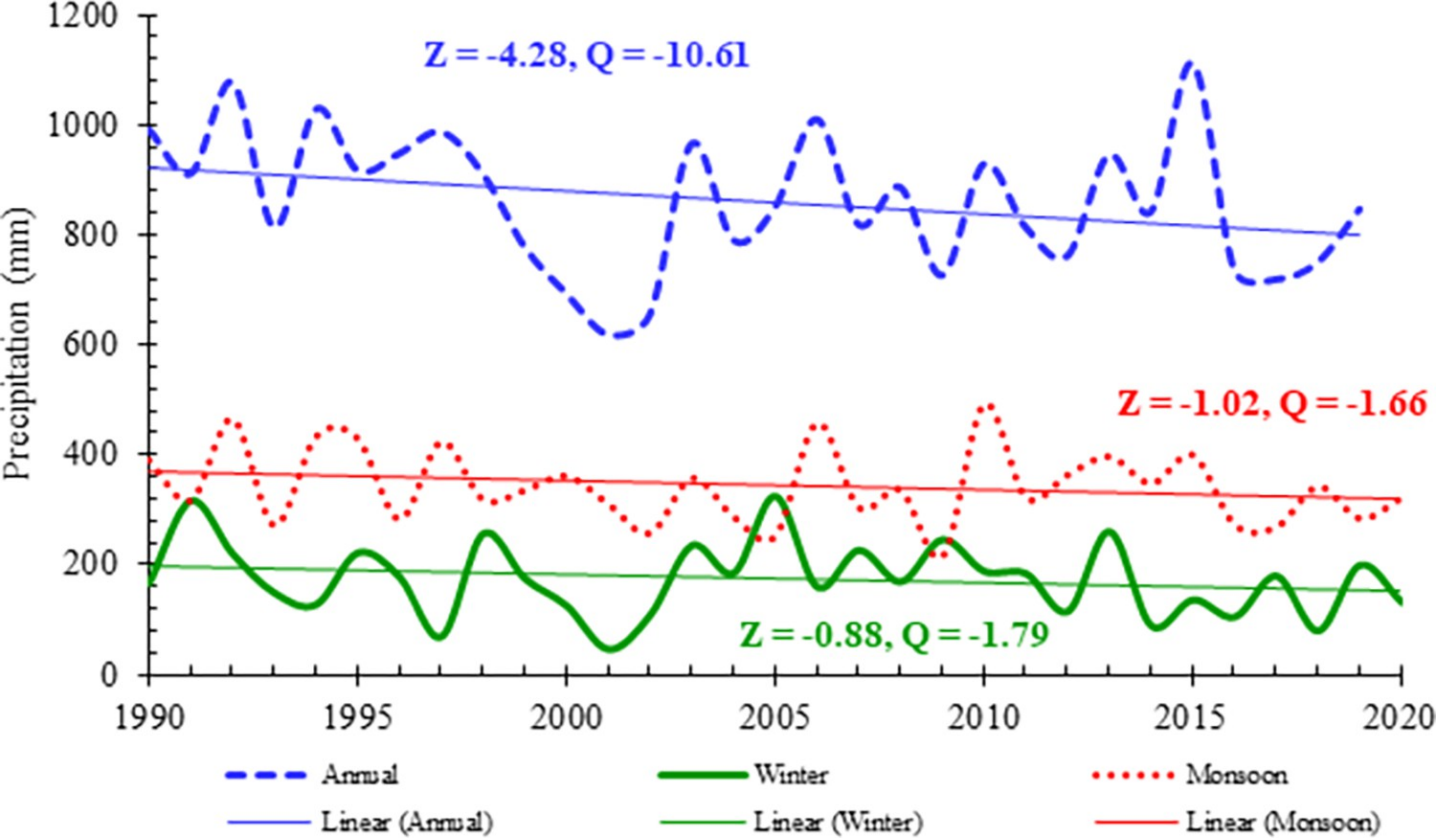
Linear trends of the total annual, winter, and monsoon precipitation in the northern high lands of Pakistan during 1990 to 2020. Z value represents the MMK test value and Q indicates the TS value.

The historical records of yearly and seasonal (winter and monsoon) precipitation for the previous 30 years were used to analyze trends in the total amount of precipitation in the NHP. These two seasons were taken into consideration based on historical precipitation records, which highlighted the prevalence of two main seasons for precipitation in the NHP. Linear trends in the total precipitation at the annual, winter, and monsoon scales are shown in Fig 3. The findings of the study showed that there has been a general trend towards less total precipitation over all time scales. The greatest rate of decrease was seen on the annual scale (-4.28 mm/year, Z = -1.50), followed by the winter season (-1.79 mm/year, Z = -0.88). The monsoon season showed a declining rate of 1.66 mm/year (Z = -1.02). Statistical analysis showed that none of these trends were significant at the 95% level.

Table 3 displays the trends of annual and seasonal total precipitation. It was found that the total annual precipitation amount (PRCPTOT) was decreased over the northern highlands of Pakistan. The amount of annual average precipitation decreased at most of the stations, and the decreasing trend at six stations was statistically significant. The rate of the decreasing trend of annual average precipitation varied from -4.44 mm/year to -19.63 mm/year. The winter and monsoon average precipitation amounts also decreased at the majority of the considered stations. The significantly decreasing rate of winter precipitation ranged from -2.29 mm/year to -9.02 mm/year, whereas the decreasing rate of monsoon precipitation ranged from -5.80 mm/year to -9.62 mm/year.

**Table 3:** Slope and trends of annual, winter, and monsoon precipitation.

Station	PRCPTOT		Winter total precipitation		Monsoon total precipitation	
	Slope	Trend	Slope	Trend	Slope	Trend
Astore	**-7.10**	**SS**	**-3.60**	**SS**	-0.27	SNS
Bagh	**-19.33**	**SS**	-4.59	SNS	**-9.62**	**SS**
Balakot	-8.93	SNS	-2.47	SNS	**-5.80**	**SS**
Bunji	0.00	SNS	0.21	SNS	0.32	SNS
Cherrat	5.00	SNS	-0.86	SNS	2.79	SNS
Chilas	-1.71	SNS	0.02	SNS	-0.72	SNS
Chitral	**-4.44**	**SS**	0.30	SNS	-0.31	SNS
Dir	-8.60	SNS	0.30	SNS	-3.90	SNS
Drosh	**-8.80**	**SS**	-1.10	SNS	-0.58	SNS
Gari Dupatta	-7.34	SNS	-3.26	SNS	-3.99	SNS
Gilgit	1.46	SNS	0.08	SNS	0.07	SNS
Gupis	-3.81	SNS	0.13	SNS	-0.55	SNS
Islamabad	1.72	SNS	0.74	SNS	-1.49	SNS
Jhelum	-10.69	SNS	-1.56	SNS	-5.90	SNS
Kakul	-3.43	SNS	-2.29	**SS**	-2.69	SNS
Kohat	4.07	SNS	-1.75	SNS	1.44	SNS
Kotli	2.43	SNS	1.06	SNS	-3.30	SNS
Muzafarabad	0.18	SNS	-4.68	SNS	6.04	SNS
Murree	**-19.63**	**SS**	**-9.02**	**SS**	**-6.77**	**SS**
Parachinar	**24.13**	**SS**	**3.75**	**SS**	5.04	SNS
Peshawar	1.63	SNS	-0.82	SNS	1.67	SNS
Rawalpindi	1.16	SNS	0.32	SNS	-4.10	SNS
Saidu Shrif	**-8.76**	**SS**	-3.33	SNS	-2.99	SNS
Skardu	-3.14	SNS	-1.93	SNS	0.13	SNS

**Fig 4:**
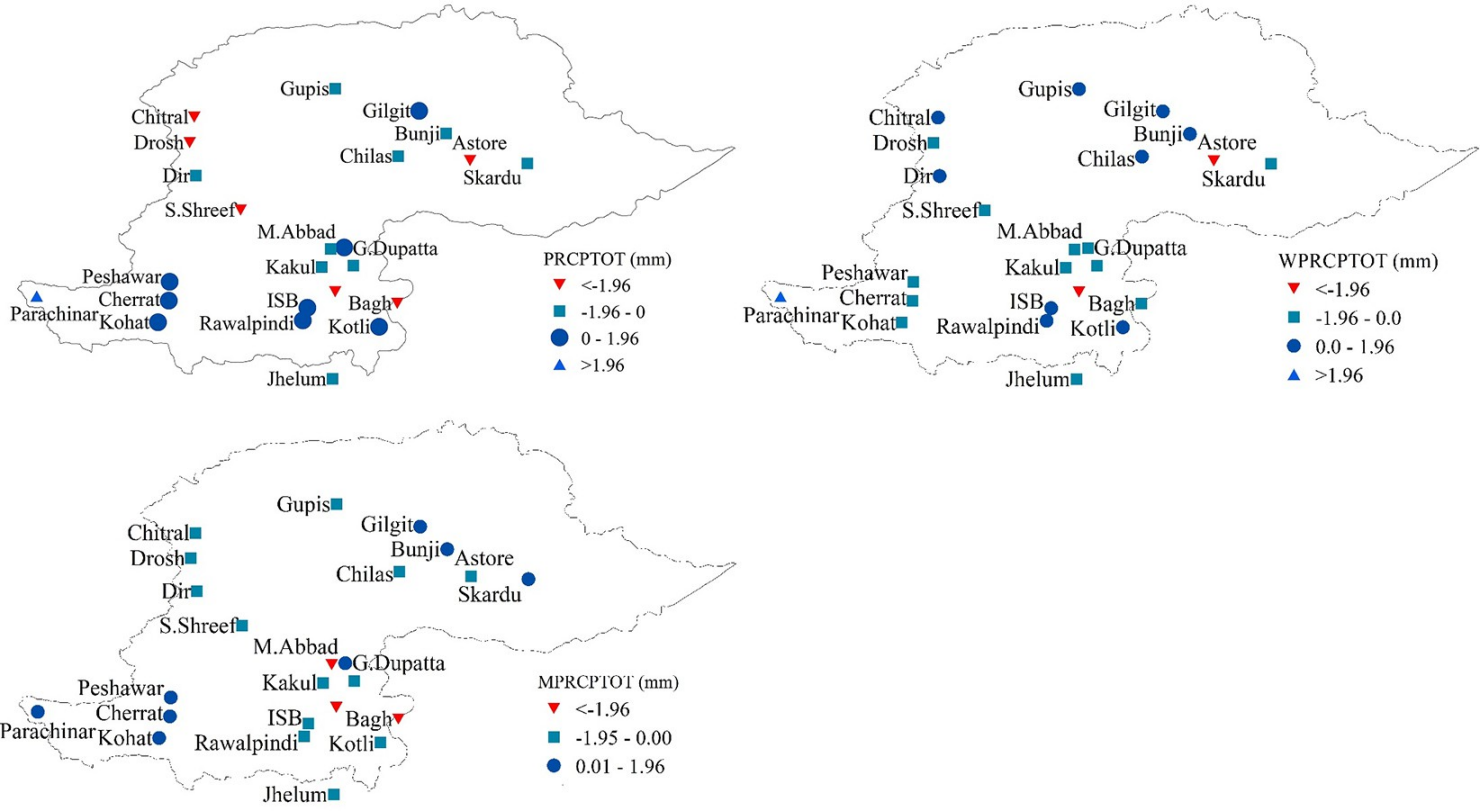
Spatial variability of MMK trend test values for annual, winter, and monsoon precipitation in the northern highlands of Pakistan.

Results indicate declining trends in total annual precipitation (PRCPTOT), winter precipitation, and monsoon precipitation across the NHP. Fig 4 displays the spatial variability of the results of MMK trend test for the total precipitation amounts at annual and seasonal scales. PRCPTOT demonstrated a decreasing trend over the northern parts of the study domain (Fig 4(A)). Six stations (out of 24) exhibited a significant decreasing trend in the PRCPTOT. Nine stations exhibited a decreasing tendency (non-significant) of total annual precipitation over the study area, whereas eight stations showed an increasing (non-significant) tendency of PRCPTOT. Only one station, Parachinar, indicated a significantly increasing trend of PRCPTOT. The overall amount of precipitation that occurs during the winter and the monsoon seasons has decreased over the course of the previous 30 years. At 14 locations, winter precipitation showed a declining trend, and statistically significant changes were detected at three stations. With the exception of Parachinar, no other station recorded a significant increase in winter precipitation. At most stations, total precipitation during the monsoon season decreased as well, with three stations spotting statistically significant decreases. Over the 30-year period, no significant positive trend was identified, despite some stations showing an increasing tendency in monsoon precipitation.

### 3.2. Temporal changes in the extreme precipitation indices

Temporal changes in the estimated precipitation indices in the NHP were analyzed using historical records of daily precipitation over the past three decades (1990–2020). The estimated extreme precipitation indices were divided in three groups depending on the intensity, frequency and dryness/wetness duration.

Table 4 shows the trends in the intensity-based precipitation indices. The R95p index showed a decreasing trend, with statistically significant trend at five stations, ranging from -9.24 to -11.76 mm/year. Only one station was experiencing an increasing trend, with an 8.67 mm/year rate. The R99p showed a significantly decreasing trend, ranging from -3.00 mm/year to -6.74 mm/year. All other trends were statistically non-significant for this index. RX1Day decreased significantly at three stations, with the change rate ranging from -1.85 to -1.89 mm/year. The RX5Day index decreased significantly at six stations, with the change rate between -1.07 mm/year and -3.61 mm/year. All other trends of RX1Day and RX5Day were statistically non-significant. SDII index was decreasing significantly at sight stations, with the rate ranging between -0.08 and -30.00.

**Table 4:** Slope and trends of intensity-based six extreme precipitation indices.

Station	R95p		R99p		RX1day		RX5day		SDII	
	Slope	Trend	Slope	Trend	Slope	Trend	Slope	Trend	Slope	Trend
Astore	-2.69	SNS	0.00	SNS	-0.32	SNS	**-1.07**	**SS**	**-0.08**	**SS**
Bagh	**-11.54**	**SS**	0.00	SNS	**-1.85**	**SS**	**-3.61**	**SS**	**-0.25**	**SS**
Balakot	**-9.24**	**SS**	**-3.00**	**SS**	**-1.89**	**SS**	**-2.68**	**SS**	**-0.12**	**SS**
Bunji	-0.79	SNS	0.00	SNS	-0.14	SNS	-0.41	SNS	-0.05	SNS
Cherrat	3.33	SNS	0.00	SNS	0.83	SNS	1.84	SNS	0.06	SNS
Chilas	0.00	SNS	0.00	SNS	0.00	SNS	-0.27	SNS	0.00	SNS
Chitral	-2.32	SNS	0.00	SNS	-0.24	SNS	-0.71	SNS	-0.03	SNS
Dir	**-9.89**	**SS**	0.00	SNS	-0.14	SNS	-1.82	SNS	**-0.10**	**SS**
Drosh	-3.41	SNS	0.00	SNS	-0.18	SNS	-0.41	SNS	**-0.13**	**SS**
Gari Dupatta	-1.61	SNS	0.00	SNS	-0.33	SNS	-1.42	SNS	0.02	SNS
Gilgit	0.23	SNS	0.00	SNS	0.15	SNS	0.00	SNS	0.00	SNS
Gupis	0.00	SNS	0.00	SNS	-0.83	SNS	**-2.00**	**SS**	**-0.30**	**SS**
Islamabad	-2.58	SNS	0.00	SNS	0.09	SNS	-0.35	SNS	-0.01	SNS
Jhelum	-5.56	SNS	0.00	SNS	-0.65	SNS	-2.34	SNS	-0.11	SNS
Kakul	**-9.24**	**SS**	-1.70	SNS	-0.34	SNS	-0.63	SNS	-0.05	SNS
Kohat	2.17	SNS	0.00	SNS	0.89	SNS	0.73	SNS	0.03	SNS
Kotli	-4.35	SNS	0.00	SNS	0.88	SNS	0.10	SNS	0.06	SNS
Muzafarabad	2.17	SNS	0.00	SNS	0.23	SNS	-1.35	SNS	**0.11**	**SS**
Murree	3.13	SNS	**-6.74**	**SS**	**-1.88**	**SS**	**-3.27**	**SS**	**-0.12**	**SS**
Parachinar	1.36	SNS	0.00	SNS	0.53	SNS	1.42	SNS	**0.17**	**SS**
Peshawar	**-11.76**	**SS**	0.00	SNS	0.04	SNS	-0.61	SNS	-0.04	SNS
Rawalpindi	**8.67**	**SS**	0.00	SNS	0.00	SNS	-1.41	SNS	0.00	SNS
Saidu Shrif	0.00	SNS	0.00	SNS	-0.35	SNS	**-2.19**	**SS**	**-0.16**	**SS**
Skardu	-2.15	SNS	0.00	SNS	0.00	SNS	0.00	SNS	0.03	SNS

“SS shows the statistically significant trend at 5% significance level.”

“SNS indicates statistically non-significant trend at 5% significance level.”

Table 5 shows the slopes and trends in two extreme precipitation indices classified based on wetness and dryness duration in a year. Overall, CDD showed a significantly decreasing trend, as revealed by a significantly decreasing trend at seven stations. The decreasing rate of CDD varies between -2.11 and -2.89 days/year. All other trends of CDD were non-significant. Similarly, CWD also showed significant decreasing trends at three stations, with the decreasing rates ranging between -0.03 and -0.11 days/year.

**Table 5:** Slopes and trends of CDD and CWD indices.

Station	CDD		CWD	
	Slope	Trend	Slope	Trend
Astore	-2.75	**SS**	0.00	SNS
Bagh	-2.45	**SS**	-0.05	SNS
Balakot	-1.67	SNS	0.00	SNS
Bunji	0.03	SNS	0.00	SNS
Cherrat	1.53	SNS	0.00	SNS
Chilas	-1.39	SNS	**-0.03**	**SS**
Chitral	-2.11	**SS**	0.00	SNS
Dir	-1.63	SNS	0.00	SNS
Drosh	-3.23	**SS**	0.00	SNS
Gari Dupatta	-1.43	SNS	-0.08	SNS
Gilgit	1.87	SNS	0.00	SNS
Gupis	-1.39	SNS	0.00	SNS
Islamabad	0.24	SNS	-0.04	SNS
Jhelum	-1.94	SNS	0.00	SNS
Kakul	-0.61	SNS	0.00	SNS
Kohat	1.02	SNS	0.00	SNS
Kotli	0.27	SNS	-0.05	SNS
Muzafarabad	-0.07	SNS	**-0.11**	**SS**
Murree	-2.89	**SS**	-0.05	SNS
Parachinar	2.48	**SS**	0.00	SNS
Peshawar	0.44	SNS	0.00	SNS
Rawalpindi	0.14	SNS	-0.04	SNS
Saidu Shrif	-2.04	**SS**	0.00	SNS
Skardu	-1.56	SNS	**-0.05**	**SS**

Table 6 displays the trends and rate of change of three extreme precipitation indices, classified based on the frequency of extreme events. Overall, all frequency-based indices showed a significantly decreasing trend in the NHP. The decreasing trend of R10 varies from -0.25 to -0.65 days/year. The R20 was decreasing, with the rate ranging between -0.09 and -0.38 days/year. In the case of R25, the decreasing rate ranged between -0.09 and -0.38 days/year.

**Table 6:** Slopes and trends of R10, R20, and R25 indices.

Station	R10		R20		R25	
	Slope	Trend	Slope	Trend	Slope	Trend
Astore	**-0.25**	**SS**	-0.08	SNS	**-0.09**	**SS**
Bagh	**-0.65**	**SS**	**-0.38**	**SS**	**-0.38**	**SS**
Balakot	-0.11	SNS	-0.12	SNS	-0.14	SNS
Bunji	0.00	SNS	0.00	SNS	0.00	SNS
Cherrat	0.13	SNS	**0.13**	**SS**	**0.11**	**SS**
Chilas	-0.08	SNS	0.00	SNS	0.00	SNS
Chitral	-0.15	SNS	**-0.09**	**SS**	**-0.09**	**SS**
Dir	-0.14	SNS	-0.21	SNS	**-0.26**	**SS**
Drosh	**-0.35**	**SS**	**-0.25**	**SS**	**-0.13**	**SS**
Gari Dupatta	-0.17	SNS	0.00	SNS	0.00	SNS
Gilgit	**0.05**	**SS**	0.00	SNS	0.00	SNS
Gupis	**-0.27**	**SS**	0.00	SNS	0.00	SNS
Islamabad	0.10	SNS	**-0.12**	**SS**	0.00	SNS
Jhelum	-0.11	SNS	0.05	SNS	-0.13	SNS
Kakul	-0.19	SNS	-0.17	SNS	0.00	SNS
Kohat	0.00	SNS	0.00	SNS	0.11	SNS
Kotli	-0.05	SNS	0.08	SNS	0.00	SNS
Muzafarabad	0.15	SNS	0.00	SNS	0.13	SNS
Murree	**-0.55**	**SS**	0.20	SNS	**-0.33**	**SS**
Parachinar	**0.71**	**SS**	-0.33	SNS	**0.33**	**SS**
Peshawar	0.00	SNS	**0.50**	**SS**	0.00	SNS
Rawalpindi	0.06	SNS	0.00	SNS	0.00	SNS
Saidu Shrif	-0.25	SNS	0.00	SNS	**-0.21**	**SS**
Skardu	0.00	SNS	0.00	SNS	0.00	SNS

### 3.3. Spatial distribution of trends of extreme precipitation indices

**Fig 5:**
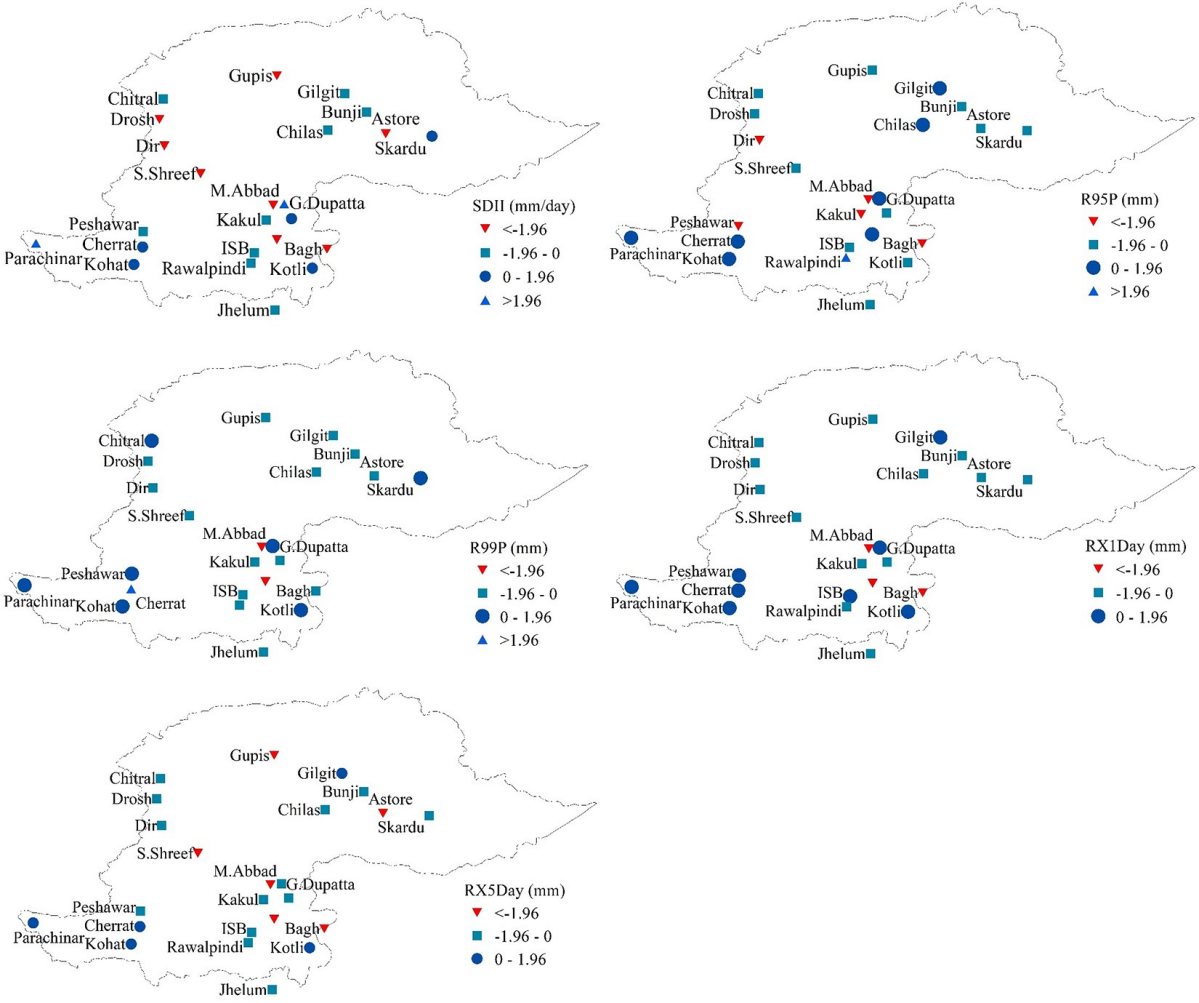
Spatial distribution of MMK trend test values for extreme precipitation indices (grouped on the basis of intensity) in the northern highlands of Pakistan.

Precipitation varies greatly across the northern highlands of Pakistan. Variability in the trends of extreme precipitation indicators (based on intensity) is shown in Fig 5. Trend analysis of the simple daily intensity index (SDII) showed an overall decreasing trend in the NHP (Fig 5 (SDII)). This index showed a statistically significant downward trend at eight sites, but a non-significant downward trend at nine stations. Even though seven (out of 24) stations exhibited an increasing trend of SDII, the increasing trend was statistically significant only at two stations. Trend analysis of R95P also revealed a decreasing pattern of very wet day precipitation in the NHP (Fig 5 (R95P). Five stations showed a significantly decreasing behavior of very wet day precipitation (R95P). In contrast, only one station in the southern area of the NHP indicated a significantly increasing trend in this index. The trend of R95P on all other stations was non-significant at 5% significant level. The trend of the R99P also indicated a decreasing pattern (Fig 5 (R99P)). Two stations indicated a significantly decreasing trend in the R99P, whereas the decreasing trend of this index on 14 stations was found non-significant at 5% significance level. Only one station exhibited a significantly increasing trend in the extremely wet day precipitation index, whereas seven stations showed a non-significant increasing tendency in this index. Trend analysis of maximum 1-day precipitation amount index (RX1day) showed an overall decreasing trend in the NHP (Fig 5 (RX1day)). Three stations indicated a significantly decreasing trend of RX1day index. The trend of this index was decreasing (non-significant) at 13 stations. Eight stations showed an increasing tendency (non-significant) of this index. No station showed a significantly increasing trend of RX1Day index. Similarly, trend analysis of the maximum 5-day precipitation amount index (RX5Day) also showed an overall decreasing trend in the NHP (Fig 5 (RX5Day)). Six stations were experiencing significantly decreasing behavior of RX5Day, whereas the decreasing trend of this index was non-significant at 13 stations. Five stations indicated a non-significant decreasing trend of RX5Day. Although five stations showed an increasing tendency of the maximum 5-day precipitation amount index, none of the considered stations indicated a significantly increasing trend of this index.

**Fig 6:**
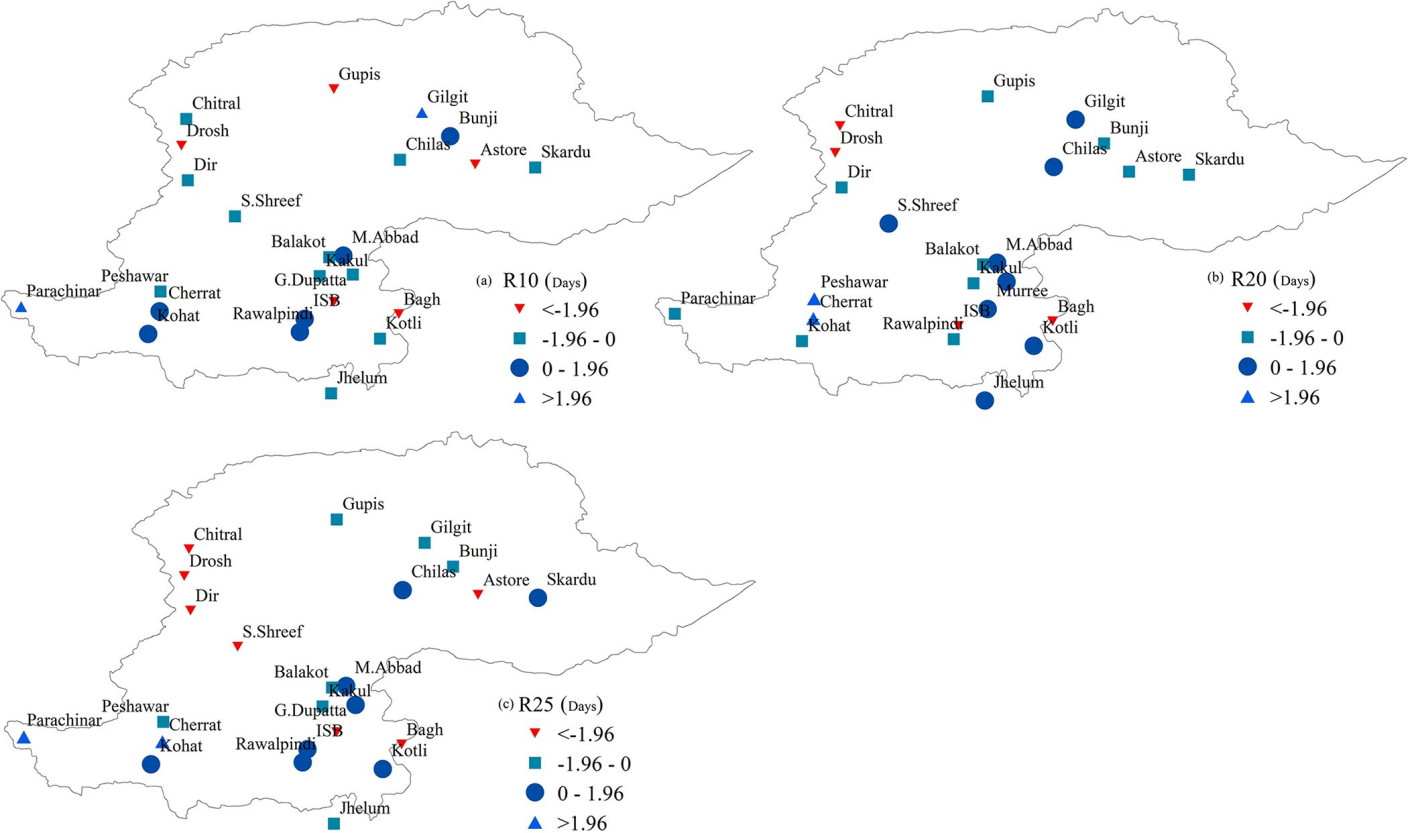
Spatial distribution of trends of R10, R20, and R24 indices in the northern high lands of Pakistan.

Fig 6 displays the spatial distribution of the trend of frequency-based indices in the NHP. Similar to the overall trend of intensity-based indices, the trend of frequency-based indices was also decreasing. Trends analysis of R10 index showed that the annual counts of days when PRCP≥10 mm was decreasing at 16 stations with a significantly decreasing trend at five stations (Fig 6 (R10)). Although this index showed an increasing behavior at eight stations, only two stations exhibited a statistically significant trend. The majority of the stations (14 out of 24) showed a decreasing trend of days in a year when PRCP≥20 mm (R20); among them, four stations experienced a significantly decreasing behavior of R20. Similarly, fourteen stations showed decreasing annual counts of days when PRCP≥25 mm (R25), with a statistically significant trend at seven stations.

**Fig 7:**
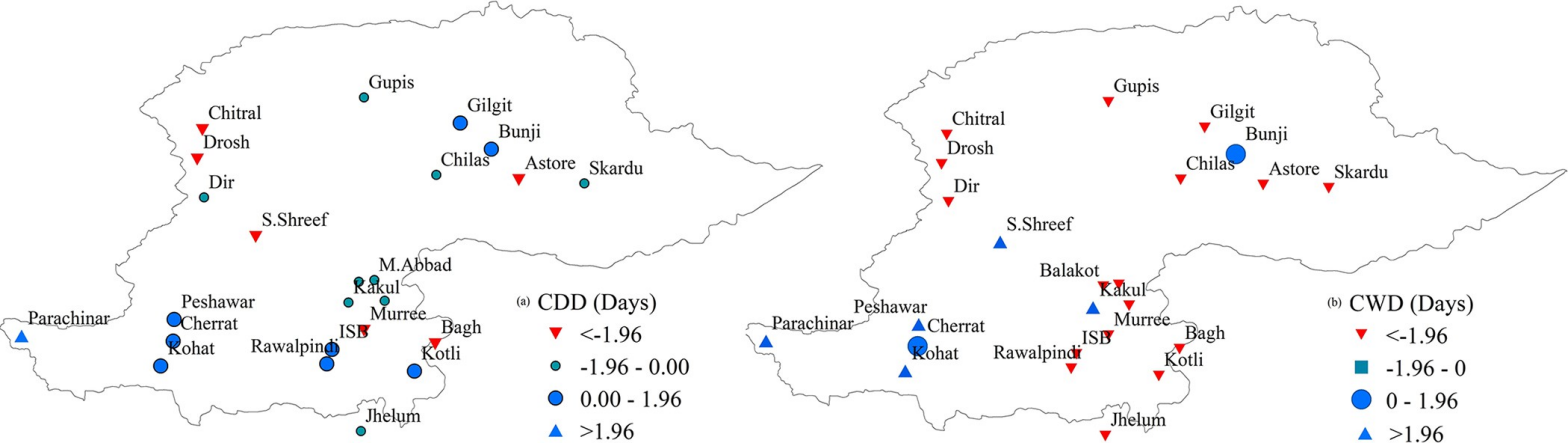
Spatial distribution of CDD and CWD trends in the northern highlands of Pakistan.

Fig 7 elaborates the spatial distribution of wetness and dryness duration based on indices. Trends analysis revealed that the CDD (precipitation <1 mm) were decreasing at majority of the stations (15 out of 24), with a significantly decreasing trend at six stations and non-significant trend at nine stations (Fig 7 (CDD)). Although nine stations indicated an increasing trend of CDD, significantly increasing trend was found only at one station. In the case of trend analysis of the CWD (precipitation ≥1 mm), it was found that the consecutive wet days were significantly decreasing in the NHP, as indicated by the decreasing trend of CWD at 17 stations (Fig 7 (CWD)). However, the southwestern part of the study area showed an increasing tendency of CWD, as witnessed by a significantly increasing trend at five stations in this part.

## 4. Discussion

For a comprehensive understanding of how climate change is affecting various parts of the world, it is critical to study the occurrence and changing characteristics of extreme precipitation events. In this study, changes in the total precipitation and 11 extreme precipitation indices are thoroughly evaluated in the NHP over a 30-year period (1990–2020). The study utilized the RClimDex model to analyze 11 extreme precipitation indices, which provides a more robust assessment of the trends and changes in precipitation patterns. Additionally, the use of MMK test and TS slope estimator to check for trends and slope of the trends offers a more in-depth and statistically significant analysis of the data.

According to the findings of the study, there is a general indication that the precipitation extreme events that have occurred in the NHP during the past 30 years has been decreasing. This is consistent with global trends of decreasing precipitation and increasing frequency of drought events [36–38]. The decreasing trend in total annual, seasonal (winter and monsoon) precipitation, intensity-based indices, and frequency-based indices indicate that the overall frequency and severity of extreme precipitation events are reducing over time and space [2, 17, 25, 30, 39]. This pattern can be related to the ongoing consequences of climate change, including increased temperatures, changing air circulation patterns, and decreased soil moisture levels [31, 40, 41]. These factors are leading to changes in precipitation patterns and intensities, with wet regions becoming drier and dry regions becoming even more arid [42].

The decreasing trend in CDD and CWD is particularly noteworthy, as it suggests that the duration of dry and wet extremes is also decreasing. This has significant implications for water resources and agriculture in the region, as extended periods of dry or wet conditions can lead to reduced crop yields and increased water stress.

The spatial distribution of the trends in the NHP highlights the heterogeneity of the region’s climate and the need for regional climate adaptation strategies. Some stations in the northern part of the study area showed a more pronounced decrease in the indices, while others showed non-significant trends or even a slight increase. These variations highlight the importance of developing regional-specific strategies for mitigation and adaptation. This results is consistent with the findings of [29, 30].

This study expands the understanding of climatic conditions that prevail in the northern Pakistani highlands. The findings of this study give an in-depth evaluation of the spatial and temporal tendencies in both total and extreme precipitation events by using the RClimDex model and conducting statistical analyses. Results indicate that downward trends are occurring across many stations in a variety of extreme precipitation indices. These indices include intensity-based indices (R95p, R99p, RX1Day, RX5Day, and SDII) as well as duration-based indices (CDD and CWD). These results add to our understanding of the climatic dynamics in this region and shed light on the effects of global warming in the subtropical highlands. These findings have important implications for the management of water resources, agriculture, and human health in the northern highlands of Pakistan. The decreasing trend in extreme precipitation events and the associated changes in the frequency and duration of dry and wet extremes call for the development of effective adaptation and mitigation strategies to ensure the resilience of communities and ecosystems under a changing climate.

We recognize that there are some limitations in our study that must be taken into account when evaluating the findings. First of all, because the research is limited to northern highlands of Pakistan, it’s possible that the results won’t be immediately transferable to other parts of the country. To have a better picture of the national patterns in Pakistan, it will be crucial for future researchers to examine the variations in extreme precipitation occurrences across the different parts of the country. Second, the time span of our study is 30 years, from 1990 to 2020. Although this time frame is sufficient for gaining useful insights into long-term trends, longer-term datasets may provide a more in-depth view of climatic fluctuations. Therefore, extending the study time would help to capture more subtle changes and improve the accuracy of the patterns that were identified.

There are several directions for future research that could build on the findings of this investigation. It would be beneficial to first examine the mechanisms responsible for the trends in extreme precipitation occurrences that have been reported in this study. Understanding the factors affecting these modifications, such as air circulation patterns, changes in land use, or aerosol effects, may shed further light on the dynamics of precipitation variability in the region. There is a need for more studies on the impact of changed trends in extreme precipitation on many sectors, including water supply, agriculture, and public health. To ensure resilience in the face of changing precipitation patterns, assessing the sensitivity and adaptive capability of communities in the northern highlands of Pakistan and designing efficient adaptation strategies is essential.

## 5. Conclusions

Based on the analysis of total (annual, winter, and monsoon) and extreme precipitation indices in the NHP, the following main conclusions can be drawn:

- The northern highlands of Pakistan (NHP) have experienced a general trend of decreased total precipitation during the past 30 years. This pattern is visible at the annual, winter, and monsoon timescales, with the annual timescale indicating the steepest rate of decrease. The rate of the decreasing trend at the annual scale varies from -4.44 mm/year to -19.63 mm/year across different stations in the NHP.
- The intensity-based precipitation indices (R95p, R99p, SDII, RX1Day, and RX5Day) have shown a decreasing trend of extreme intensity events over time, with statistically significant trends at multiple stations.
- The CDD (number of consecutive dry days) and CWD (number of consecutive wet days) indices have shown a significant decreasing trend at multiple stations. The decreasing rate of CDD varies between -2.11 and -2.89 days/year, while the decreasing rate of CWD varies between -0.03 and -0.11 days/year. These results suggest that the duration of both dry and wet extremes is decreasing over time.
- The number of days in a year with precipitation larger than or equal to 10 millimetres (R10), 20 millimetres (R20), and 25 millimetres (R25) were decreasing at the majority of the considered stations. The rate of change ranges from -0.25 to -0.65 days/year for R10, -0.09 to -0.38 days/year for R20, and -0.09 to -0.38 days/year for R25. These results suggest that the frequency of extreme precipitation events is decreasing over time and space.
- Spatial variability of the MMK test results shows that the decreasing trend is more pronounced in the northern parts of the NHP, while some stations indicated non-significant decreasing or increasing trends. However, only a few stations exhibited a significant increasing trend in the indices.

Based on the findings of this study, it can be established that there is generally a decreasing trend in the total annual precipitation and various extreme precipitation indices across the northern highlands of Pakistan. However, the trends vary by region, with some stations experiencing significant decreases and others showing increasing or non-significant tendencies. It is important to note that these results are based on data from specific stations in the NHP region, and the trends may differ in other parts of Pakistan. Further research is needed to establish a more comprehensive understanding of the trends in extreme precipitation events across the country.
